# A Review of Epithelial Ion Transporters and Their Roles in Equine Infectious Colitis

**DOI:** 10.3390/vetsci11100480

**Published:** 2024-10-07

**Authors:** Lillian M. B. Haywood, Breanna J. Sheahan

**Affiliations:** Department of Clinical Sciences, College of Veterinary Medicine, North Carolina State University, Raleigh, NC 27607, USA; lmhaywoo@ncsu.edu

**Keywords:** equine, gastrointestinal, colitis, diarrhea, ion channels, infectious disease

## Abstract

**Simple Summary:**

Equine colitis, or inflammation of the colon of the domestic horse, can occur rapidly and lead to death within hours to days. It is often associated with severe diarrhea leading to dehydration. There are many different causes of colitis, including infections with bacteria, viruses, and parasites, as well as non-infectious causes. Antimicrobial administration can be associated with the development of infectious colitis, likely because of disrupted intestinal microbiota allowing for the expansion of pathogenic bacteria. Ion channels regulate electrolyte and fluid movement into and out of the colon lumen. These channels can be manipulated by different infectious organisms to drive increased fluid movement into the intestinal lumen. Current treatments for equine colitis are untargeted and largely supportive in nature. In other species, targeting ion channels has emerged as a potential treatment for diarrhea. This may represent a novel avenue for anti-diarrheal therapies in the horse. However, ion channels have not been well studied in the colon of the horse. This review provides an overview of what is known about colonic ion channels and their known or putative role in specific types of equine colitis due to various pathogens.

**Abstract:**

Equine colitis is a devastating disease with a high mortality rate. Infectious pathogens associated with colitis in the adult horse include *Clostridioides difficile*, *Clostridium perfringens*, *Salmonella* spp., *Neorickettsia risticii*/*findlaynesis*, and equine coronavirus. Antimicrobial-associated colitis can be associated with the presence of infectious pathogens. Colitis can also be due to non-infectious causes, including non-steroidal anti-inflammatory drug administration, sand ingestion, and infiltrative bowel disease. Current treatments focus on symptomatic treatment (restoring fluid and electrolyte balance, preventing laminitis and sepsis). Intestinal epithelial ion channels are key regulators of electrolyte (especially sodium and chloride) and water movement into the lumen. Dysfunctional ion channels play a key role in the development of diarrhea. Infectious pathogens, including *Salmonella* spp. and *C. difficile*, have been shown to regulate ion channels in a variety of ways. In other species, there has been an increased interest in ion channel manipulation as an anti-diarrheal treatment. While targeting ion channels also represents a promising way to manage diarrhea associated with equine colitis, ion channels have not been well studied in the equine colon. This review provides an overview of what is known about colonic ion channels and their known or putative role in specific types of equine colitis due to various pathogens.

## 1. Introduction

The horse has a unique gastrointestinal tract that is characterized by an enormous cecum and colon. The equine cecum and colon comprise the large intestine and are responsible for two major functions: microbial digestion (fermentation) and the storage and absorption of large volumes of water [1]. This anatomy is markedly different from other herbivores, especially ruminants; the equine stomach is relatively small and has no fermentation role, while the cecum can hold up to 30 L of ingesta and the colon 80 L [2]. It is estimated that the equine large intestine secretes and resorbs approximately 100 L of fluid each day, a volume equivalent to the horse’s entire extracellular volume [3,4]. The volume and surface area of the hindgut in the horse makes it especially susceptible to rapid and deadly fluid loss secondary to the development of diarrhea [5]. Due to the considerable volume of the equine large intestine, colonic inflammation and subsequent diarrhea quickly results in net fluid loss, electrolyte imbalances, and hypovolemia.

Because of these serious sequelae, equine diarrhea is responsible for between 0.7 and 5 percent [6] of hospital admissions, with mortality estimates ranging from 20 to over 50 percent [7,8,9]. Bacterial and viral pathogens known to cause colitis in adult horses include *Clostridioides difficile*, *Salmonella* spp., Potomac Horse Fever (*Neorickettsia risticii*/*Neorickettsia findlaynesis*), and equine coronavirus. *Clostridium perfringens* is most commonly associated with colitis in foals but has been associated with colitis in adult horses. Colitis can also be due to parasitic infections, specifically the emergence of encysted small strongyles (cyathostomins). Antimicrobial administration is associated with the development of colitis, often concurrently with infection with *Salmonella* spp. and *C. difficile* [10]; it is suspected that this is due to the changes in the normal gastrointestinal flora induced by antimicrobial administration [11,12]. Non-infectious causes of equine colitis include non-steroidal anti-inflammatory drug administration, inflammatory bowel disease, neoplasia, and sand enteropathy [13,14]. However, despite improved diagnostics, over 50 percent of equine colitis cases do not have an etiology identified (undifferentiated colitis) [15]. This review will focus on infectious causes of diarrhea in the horse, including a brief discussion of antibiotic-associated diarrhea, and what we know about the pathophysiology of each pathogen as it relates to ion and fluid transport. Because the horse has a unique gastrointestinal tract as compared to other species studied, data extrapolated from humans and lab animal species may not be directly translatable to the horse. However, due to limited equine-specific studies, knowledge gained from other species will be discussed, particularly in relation to the regulation of ion channels by pathogens. This limited equine knowledge underscores the need for further studies using equine-specific models.

## 2. Anatomy of the Intestinal Epithelium

**Figure 1:**
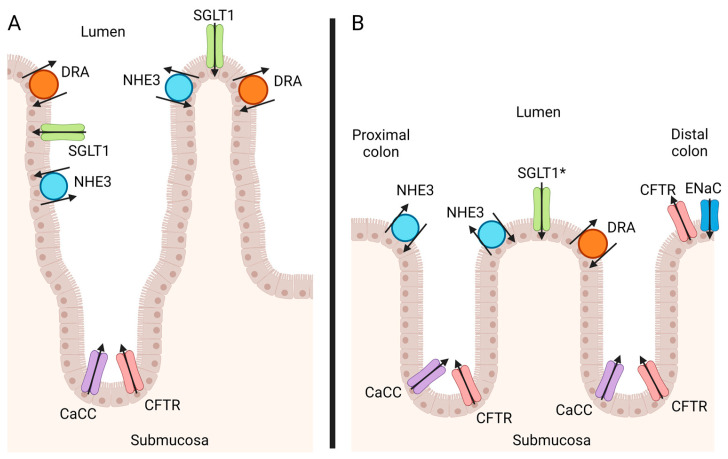
Localization of epithelial apical ion channels along the intestinal tract. (**A**) The epithelium of the small intestine is separated into crypt and villus regions, with absorptive ion channels (including NHE3, SGLT1, DRA) located along the villus tip and secretory ion channels (including CFTR, CaCC) within the crypt. (**B**) The colonic epithelium has secretory ion channels (including CFTR, CaCC) within the crypts and absorptive ion channels (including NHE3, SGLT1, DRA, ENaC) located at the mucosal surface. In the horse, DRA (anion-exchanger) is present on both the right ventral and right dorsal colons. Bicarbonate is also secreted via CFTR in the horse (primarily in the right dorsal colon). ENaC is more highly expressed in the distal colon of rodents and humans (and can be co-localized with CFTR), while NHE3 tends toward higher expression in the proximal colon of rodents and humans. Equine distribution of ENaC, NHE3, CFTR, and CaCC is unknown. (*) SGLT1 is present at low levels in colonic epithelium in rodents and humans. CaCC: Calcium-activated chloride channel; CFTR: Cystic fibrosis transmembrane conductance regulator; ENaC: Epithelial sodium channel; SGLT1: Sodium-glucose cotransporter 1; NHE3: Sodium-hydrogen antiporter 3; DRA: Down regulated in adenoma (also known as the anion exchanger). Image created with Biorender.

The colonic and cecal microanatomy consist of a single layer of epithelial cells, which are organized into crypts and a relatively flat mucosal surface. The differentiated epithelial cells largely consist of absorptive enterocytes and goblet cells, with stem cells residing in the crypt bases. In the small intestine, the differentiated epithelium is present on long, finger-like projections called villi, and the absorption of ions, proteins, glucose, and other molecules primarily occurs across this differentiated epithelium (Figure 1A). In the colon, secretion occurs primarily in the crypt epithelium, and absorption on the flat mucosal surface at the top of the crypts (Figure 1B).

It is hypothesized that the secretory function of the crypt cells in both the small and large intestine is important to maintain the crypt base (and resident intestinal stem cells) in a sterile environment, by flushing out the crypts through secretory action [16]. Much of this action in the colon is achieved through ionocytes or deep crypt secretory cells, which in addition to secreting electrolytes and producing mucins, also produce growth factors necessary for stem cell proliferation [17].

Diarrhea can arise from altered transcellular mechanisms of ion and fluid transport, as well as the loss of epithelial barrier integrity. The ion transport mechanisms that are targeted by various intestinal pathogens will be discussed in further detail in this review. There are two broad categories of diarrhea due to altered transcellular ion transport—secretory and osmotic/malabsorptive. In secretory diarrhea, active secretion of chloride exceeds absorption [18] and provides an osmotic basis for water to follow [16]. In most cases of malabsorptive diarrhea, the epithelial cells fail to absorb sodium and/or other molecules, again providing an osmotic basis for water to remain in the colonic lumen [19]. The relative contribution of these mechanisms to diarrhea has not been fully elucidated in the horse.

## 3. Epithelial Ion Channels in the Equine Colon

The fluid and electrolyte balance that takes place in the colon requires several epithelial ion channels, organized at three levels: intracellularly (on the basolateral or apical surface of the cell membrane), along the crypt/mucosal surface (colon) axis, and along the length of the gastrointestinal tract [16] (Figure 1). The key ion involved in fluid balance is chloride [16], but sodium, bicarbonate, and potassium ions also play critical roles. There is limited knowledge regarding the expression, distribution, and activity of ion channels in the intestinal epithelium of the horse. Therefore, much of the current knowledge regarding ion channels and their organization has been generated in other species. These will be discussed as broadly applicable across species, as many of the ion channels are conserved between species, but the reader should keep in mind that most of this has not been directly studied in the horse. Horse-specific research will be denoted where appropriate.

### 3.1. Chloride

Chloride ions play the largest role in fluid balance in the colon in most mammalian species. These ions can be transported via both passive and active means, via channels located both apically and basolaterally on colonic epithelial cells. Chloride is maintained intracellularly at relatively high concentrations compared to extracellular levels. This is accomplished through the combined efforts of two basolateral ion channels: the sodium–potassium ATPase pump (Na^+^/K^+^ ATPase) and the sodium–potassium–chloride cotransporter pump (NKCC1). The energy-requiring Na^+^/K^+^ ATPase pump actively pumps sodium ions out of the cell and potassium ions into the cell, against their osmotic gradients. Therefore, when the NKCC1 transporter is opened, it allows sodium ions to flow down their concentration gradient, into the cell, co-transporting chloride ions into the cell alongside the sodium and potassium ions. NKCC1 is expressed throughout the gastrointestinal tract, with the highest expression in the colonic crypts [16,18].

**Figure 2:**
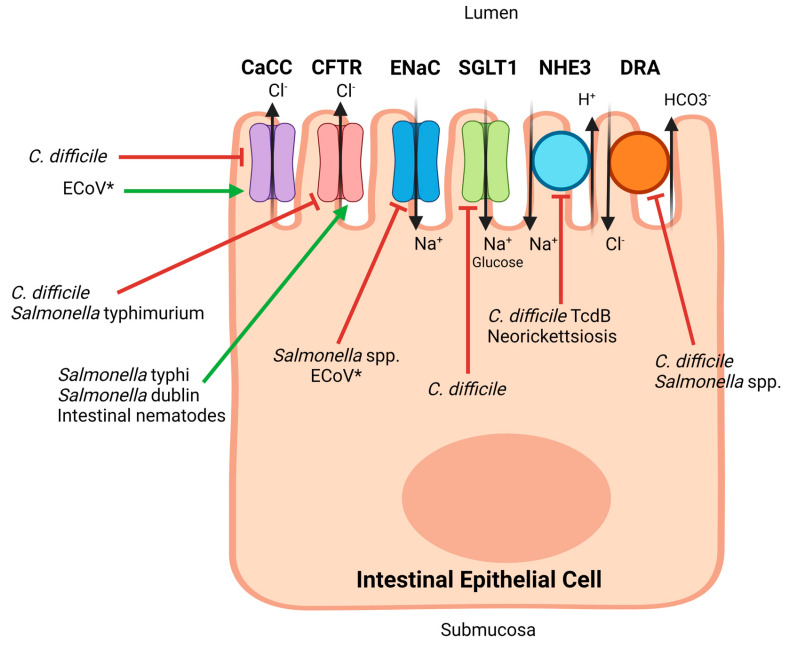
Absorptive and secretory ion channels of the intestinal epithelium and their regulation by pathogens or pathogen-secreted toxins. The red bars indicate inhibition of the targeted channel, while the green arrows indicate activation. The figure is not intended to imply that the ion channels are all expressed in the same cell but rather to demonstrate the potential conflicting influences of different pathogens on epithelial ion transport. References for each proposed target by the different pathogens are included in Table 1. (*) The proposed mechanism of action of ECoV is extrapolated from data on related pathogens (SARS-CoV and SARS-CoV-2). CaCC: Calcium-activated chloride channel; CFTR: Cystic fibrosis transmembrane conductance regulator; ENaC: Epithelial sodium channel; SGLT1: Sodium-glucose cotransporter; NHE3: Sodium-hydrogen antiporter 3; DRA: Down regulated in adenoma (also known as the anion exchanger), TcdB: Toxin B. Image created with Biorender.

On the apical membrane of colonocytes, with its highest expression in colonic crypts [18], is the cystic fibrosis transmembrane conductance regulator (CFTR) channel (Figure 2). When activated, CFTR allows chloride to flow down its concentration gradient into the colonic lumen. The opening of CFTR is induced via the cyclic AMP (cAMP) or cyclic GMP (cGMP) second messenger pathways, which are common downstream mediators of several different pathogens [20]. For example, in humans, CFTR activation can occur via the binding of cholera toxin to the GM1 receptor, inducing cAMP production, or via the binding of heat-stable enterotoxin (STa) to the guanylin receptor, inducing cGMP production [21]. Cholera toxin is able to permanently alter GM1 so that it is continually activated, resulting in the continual production of cAMP and chloride secretion, resulting in severe diarrhea [22]. Natural *Vibrio cholerae* infection has rarely been reported in horses [23], but when cholera toxin is applied to equine colonic tissue, there is a significant increase in cAMP, indicating that this pathway is intact in the horse [24].

cAMP production can also be induced via the activation of enterochromaffin cells, resulting in the production of vasoactive intestinal peptide (VIP) and substance P, inflammatory mediators [21], and prostaglandins [25]; these are likely the mechanisms through which CFTR activation occurs in the horse. In horses, VIP has been localized to the jejunum, ileum, cecum, and colon’s associated neuronal structures [26], and both VIP and substance P levels are altered in the equine colon during ischemia-reperfusion [27]. In a human cell line, it was demonstrated that prostaglandin F_2_ is as effective at activating CFTR as forskolin, a potent cAMP stimulator. In the horse, prostaglandin F_2a_ is produced in response to endotoxemia [28], which is a common sequelae of colitis, and another prostaglandin, prostaglandin E_2_, is produced by the cecum in an experimental colitis model [29].

Calcium-activated chloride channels (CaCCs) may also play a role in chloride secretion on the apical surface of enterocytes. As the name implies, CaCC receptors are activated via the utilization of calcium as a secondary messenger [21]. In mice, the rotavirus toxin NSP4 is able to activate the CaCC receptor via calcium signaling, thus inducing active chloride secretion and secretory diarrhea [18]. These receptors likely play a role in chloride secretion in cases of cystic fibrosis (where patients have a mutated and therefore a partially or fully non-functional CFTR) and in young animals [18]. This may partially explain the differences in rotavirus pathogenicity between adults and young animals.

### 3.2. Sodium

The epithelial sodium channel (ENaC) is the most important sodium channel in the distal colon and rectum, where it is responsible for the electrogenic absorption of sodium, which is followed by chloride and water (Figure 2). CFTR channels are also found in colonocytes at the mucosal surface near the top of the crypts; when in this location, they are generally colocalized with ENaC channels [18] (Figure 1B). CFTR regulates the function of ENaC inversely—when CFTR is activated, ENaC is deactivated [30]. This inhibition of ENaC during the activation of CFTR appears to be associated with a decrease in channel open probability for ENaC, not a decrease in the expression of the ion channel [31], but the mechanism by which the CFTR and ENaC channels interact is still unclear [32]. ENaC functionality is also controlled by the presence of serine proteases, which may play a role in inflammatory bowel disease [33]. Lastly, ENaC is aldosterone-responsive: aldosterone is able to regulate ENaC activity by controlling the number of receptors present on the apical surface, and the probability that the channels are open [34]. In the horse, exogenously administered aldosterone induced increased sodium absorption in the ventral, dorsal, and small colon, correlating with ENaC expression along the colon [35]. Clinically, circulating aldosterone concentrations increase with signs of dehydration (increased packed cell volume and creatinine) and sodium depletion [36], common sequelae of diarrhea in the horse. The effect of circulating aldosterone on ENaC function in an inflamed equine colon is unknown.

Another significant sodium channel is the sodium/hydrogen exchanger (NHE), which is responsible for the electroneutral exchange of sodium and hydrogen ions. Of the eight described NHE isoforms, NHE3 plays the most significant role in sodium/hydrogen exchange in the colon (Figure 2). It may also play a role in chloride secretion and, in some cases, may be inversely associated with CFTR activation [37]; in the kidney, sodium absorption by NHE is inhibited by the cAMP secondary messenger pathway in direct opposition to CFTR [38]. NHE3 may also be associated with the chloride/bicarbonate exchanger (downregulated in adenoma or DRA) and may be activated by short-chain fatty acids [39]. In both a human and rat model, the short-chain fatty acids butyrate, acetate, propionate, and isobutyrate all increased the expression and activity of NHE3, but not NHE2 [40]. In the horse, short-chain fatty acids are produced during fiber fermentation by commensal bacteria in the large intestine, and are utilized by the horse for 70–80 percent of its energy requirements [41,42]. Therefore, if the colonic microbiome is disrupted, such as through antimicrobial administration, short-chain fatty acid production is inhibited, and NHE3 function may be affected.

The sodium-glucose cotransporter (SGLT1) also plays a role in sodium absorption, especially in small intestinal enterocytes, where the majority of glucose absorption occurs [43] (Figure 2). Cholera-associated diarrhea can be managed with the manipulation of this cotransporter—when glucose is added to electrolyte (sodium)-rich oral fluids, oral rehydration is significantly more effective [44]. On the basolateral surface of colonocytes, the previously mentioned Na^+^/K^+^ ATPase pump and NKCC1 channel also play a role in sodium transport.

### 3.3. Potassium

The colonic epithelial potassium channels are relevant for the management of water, sodium, and chloride balance, especially the ability of the animal to secrete chloride into the colonic lumen [45]. Potassium ions are transported into the colonic epithelial cell electrogenically via the Na^+^/K^+^ ATPase pump, and electroneutrally via the NKCC1 cotransporter. The two basolateral potassium channels are KCNQ1, a voltage-gated channel activated by cAMP, and KCNN4, a channel which is activated by intracellular calcium [20]. KCNQ1 activation is closely associated with chloride secretion during CFTR activation; when KCNQ1 is inactivated, electrogenic chloride secretion almost completely halts [45]. The transport of potassium ions into the epithelial cell plays a critical role in maintaining an electrochemical gradient across the colonic epithelium, which is required for the electrogenic transport of electrolytes [45]. In particular, potassium channels are critical in maintaining high intracellular levels of chloride ions, which can subsequently be secreted apically via the activation of CFTR [20]. Potassium channels in the colon are also activated by aldosterone, allowing for the movement of potassium down its concentration gradient and into the gastrointestinal lumen for excretion by the organism [45].

### 3.4. Bicarbonate

In colonocytes, bicarbonate accumulates intracellularly either via cellular metabolism or a coupled sodium/bicarbonate transporter located on the basilar cell membrane [46,47]. Apical bicarbonate exchange in the guinea pig, rabbit, rat [37], and horse [48] occurs via three main mechanisms: electrogenic efflux through the CFTR receptor, the apical chloride/bicarbonate exchanger (DRA) (Figure 2) and a short-chain fatty acid/bicarbonate exchanger [37]. All three mechanisms result in the secretion of bicarbonate into the colonic lumen; once in the lumen, bicarbonate serves as a critical buffer to protect the mucosa and fermentation process [48]. In the horse colon, there are regional differences in bicarbonate secretion through CFTR but not DRA [48]. The horse’s right dorsal colon has a greater bicarbonate secretion via CFTR (as compared to the right ventral colon), and CFTR is activated by prostaglandins. This may explain why the right dorsal colon is uniquely sensitive to the effects of non-steroidal anti-inflammatory drugs [49]. As prostaglandin formation is blocked by non-steroidal anti-inflammatory drugs (NSAIDs), these drugs are able to block bicarbonate secretion and subsequently reduce the buffering capacity within the right dorsal colon [48,50]. Loss of this buffer leads to mucosal damage and secondary right dorsal colitis.

## 4. The Contributions of Secretory versus Malabsorptive Mechanisms in the Horse

Herbivores secrete copious volumes of fluid in their upper gastrointestinal tract to aid in digestion. In a 100 kg pony, the secretions from the parotid gland, biliary system, and pancreas total 30 L per day; the small intestine and stomach secrete further, albeit unknown, quantities of fluid into the gastrointestinal tract [51]. The majority of this fluid, as well as any liquids ingested, makes its way to the large intestine, where it is reabsorbed. Therefore, it stands to reason that in horses (as with most herbivores), simply failing to reabsorb this fluid can rapidly result in severe dehydration and prove fatal [51]. Consistent with this, there is evidence that sodium may be the most important ion in maintaining fluid balance in horses. Argenzio et al. in 1975 showed that sodium ion flux was most closely related to large intestinal water movement in healthy ponies [1]. Further, Ecke et al. in 1998 showed that in an experimental castor oil model of equine diarrhea, the onset of clinical diarrhea correlated closely with increased fecal dry matter sodium concentration and no change in fecal chloride concentration [52]. These two studies imply that diarrhea in horses may be primarily due to decreased sodium absorption (via ENaC and NHE) rather than increased chloride secretion, but molecular studies examining the differences in ion channels between healthy and colitis-afflicted horses have not yet been performed.

Clinically, decreased absorption can occur via pathogens that induce the cell death of epithelial cells, leading to a loss of the absorptive surface area (such as rotavirus and equine coronavirus, which cause blunting of the villi in the small intestine [53]) and the downregulation of ion channels located on the microvilli of epithelial cells (such as occurs with *Neorickettsial* disease [54]) [51]. This is in contrast to the traditional definition of malabsorptive diarrhea, where high solute concentrations, such as the high levels of lactose in milk replacer or from dams overproducing milk, cause osmotic diarrhea [55].

However, studies in humans and in laboratory animals have shown that many pathogens (including *E. coli*, *Shigella flexneri*, and *Vibrio cholerae*) cause an active secretion of fluid [18]. This secretion occurs through a variety of mechanisms, including an alteration in ion transport (especially due to cAMP generation by enterotoxins, leading to CFTR activation), tight junction disruption, effects on the enteric nervous system, and increased blood flow to the colon, due to the production of nitrous oxide and vasoactive intestinal peptide as part of the host’s inflammatory response [18]. Of these mechanisms leading to increased secretion, the activation of CFTR is particularly important; humans and mice that lack a functional CFTR are resistant to the effects of many diarrheal pathogens. The role of CFTR in equine colitis has not been fully elucidated, but one study demonstrated a significant increase in cAMP production when cholera toxin was applied to the equine colon [24], thus suggesting that the pathway for CFTR activation due to infectious pathogens exists in equine colonocytes.

Extrapolating from other species and the earlier studies from Argenzio and Ecke, it is expected that both active secretion and failure of reabsorption play roles in equine colitis [1,52]. Furthermore, it has been demonstrated in other species that different pathogens are able to activate and inactivate a unique pattern of ion channels, which presumably also occurs in the horse but is difficult to differentiate clinically (Figure 2). For example, in murine models, a toxin associated with *Clostridioides difficile* was shown to inhibit sodium absorption through NHE3, while *Salmonella* was shown to inhibit ENaC [56]. There are few studies critically evaluating the pathogen manipulation of ion channels in diarrheal diseases of horses, and the majority of proposed mechanisms discussed in the next section are extrapolated from other species (Table 1). Due to the horse’s unique intestinal anatomy, findings from other species should be interpreted with caution.

**Table 1:** Pathogens associated with enteric infections in the adult horse, the pathogen’s effect on ion transport, and the animal or cell model demonstrating the effect. Equine-specific models are noted in bold. Animal models noted as “ex vivo” generally suggests functional testing, such as Ussing chambers, shortly after tissue removal from the animal or human. CaCC: Calcium-activated chloride channel; CFTR: Cystic fibrosis transmembrane conductance regulator; ENaC: Epithelial sodium channel; SGLT1: Sodium-glucose cotransporter 1; NHE3: Sodium-hydrogen antiporter 3; DRA: Down regulated in adenoma (also known as the anion exchanger).

Pathogen	Serovar	Toxins Produced	Ion Transporter Affected	Absorption or Secretion	Animal/Cell Model	Reference
*Clostridioides difficle*		TcdA and TcdB	Decrease CFTR function	Decrease secretion–chloride	In vivo and ex vivo murine colon	[57]
			Decrease CaCC function	Decrease secretion–chloride	In vivo and ex vivo murine colon	[57]
			Decrease DRA expression	Decrease absorption–chloride	In vivo and ex vivo murine colon	[57]
			Decrease SGLT1 expression	Decrease absorption–sodium, glucose	In vivo and ex vivo murine colon	[57]
		TcdB only	Decrease NHE3 expression	Decrease absorption–sodium	In vivo and ex vivo murine colon	[57]
*Clostridium perfringens*		CPE	N/A—Tight junction disruption, pore formation	Passive loss into lumen	Rabbit ileum, Vero kidney cell line (African green monkey)	[58,59]
		CPB2	N/A—Pore formation	Passive loss into lumen	Murine (intravenous administration), I407 cell line (human), rabbit small intestine and colon, **ovarian tumor cell line (equine)**	[60,61,62]
		CPB	N/A—Endothelial damage	N/A	Neonatal porcine small intestine	[63]
*Salmonella* spp.	Typhimurium		Decrease ENaC expression and activity	Decrease absorption–sodium	Ex vivo murine colon	[64]
	Typhimurium		Decrease CFTR function	Decrease secretion–chloride	Ex vivo murine colon	[64]
	Typhi		Increase CFTR function	Increase secretion–chloride	Murine small intestine, T-84 cell line (human)	[65]
	Dublin		Increase CFTR function	Increase secretion–chloride	HT-29, Caco-2, T-84 cell lines (human)	[66]
	Typhimurium		Decrease DRA expression	Decrease absorption–chloride	Murine colonic organoids	[67]
Neorickettsiosis			Decrease NHE3 activity (suspected to also occur in the horse)	Decrease absorption–sodium	**Equine colon**, T-84 cell line (human)	[54,68]
Equine Coronavirus			N/A—Villus blunting	Decrease absorption–sodium, glucose	**Equine small intestine**	[53]
			Increase CaCC activity (demonstrated in SARS-CoV2)	Increase secretion–chloride	Ex vivo human colonic mucosa (SARS-CoV2)	[69]
			Decrease ENaC activity (demonstrated in SARS-CoV)	Decrease absorption–sodium	Expression of human ENaC in *Xenopus* oocytes (SARS-CoV)	[70]
Cyathostomins			N/A—Mucosal surface disruption	Passive loss into lumen	**Equine colon**	[71]
			N/A—Increase mucin production	N/A	**Equine enteric monolayers**	[72]
			Increase CFTR or CaCC function (demonstrated in other intestinal nematodes)	Increase secretion–chloride	Ex vivo rat colon	[73]

## 5. Clinical Overview of Colitis Pathogens in the Horse

There have been numerous retrospective studies on equine colitis at tertiary care centers in the literature. It can be difficult to compare studies between hospitals due to how colitis is defined, the regionality and seasonality of Neorickettiosis and *Salmonella* spp. [74], clinician preference on diagnostic testing and treatment, and owner economic constraints [14]. In areas where Neorickettsiosis is endemic, those cases make up a significant portion of cases [75,76] and also represent a significant portion of the cases that do not make it to discharge, likely due to the pathogen’s association with laminitis [76]. Other factors associated with a poor prognosis include *Clostridioides difficile* infection [8], length of hospitalization [7] and length of time between onset of clinical signs and referral [77], the age of the horse, a history of antimicrobial usage, physical exam findings such as colic signs and increased heart rate, and clinical pathology findings, such as increased packed cell volume, increased creatinine, and decreased total protein [8,78,79]. The published survival rates of horses with colitis vary from 49 percent [9] to 76 percent [80].

Clinical signs at admission can vary but most commonly include fever, colic signs, anorexia, dehydration, clinical endotoxemia, and diarrhea [80,81]. However, an absence of diarrhea does not rule out colitis—in one study, only 8 out of 42 horses diagnosed with colitis presented with abnormal fecal character [81]. Common clinical pathological abnormalities include leukopenia [82], hyponatremia, hypokalemia, hypochloremia, hyperlactatemia, and hypoproteinemia [80]. Common sequelae of colitis include laminitis [8,9,75], disseminated intravascular coagulation (DIC) [83], and thrombophlebitis [8].

### 5.1. Clostridioides difficile

*Clostridioides difficile*, previously known as *Clostridium difficile*, is the leading cause of nosocomial gastroenteritis in humans [84] but is also an important cause of enterocolitis in the horse. When acute colitis cases are tested for pathogens, between 8 and 25 percent of horses test positive for *C. difficile* [8,14]. Horses with diarrhea that are positive for *C. difficile* have a higher mortality rate than horses with diarrhea that are negative [85]; in one study, horses with *C. difficile*-associated diarrhea (CDAD) were 2.7 times more likely to die or be euthanized [14]. The largest risk factor for CDAD in horses is antimicrobial administration [14,86,87]. Classically, *C. difficile* colonizes the cecum and colon, causing a pseudomembranous colitis [88], but it has been associated with anterior enteritis [89], and foals may also develop small intestinal lesions^,^ [90]. Clinically, CDAD is not highly specific and resembles other causes of equine colitis, with diarrhea, fever, and signs of colic as the most common clinical signs [90,91,92]. Disease severity can range from moderate disease to a rapidly fatal peracute colitis [6], with metronidazole-resistant strains seeming to be more virulent [93].

*C. difficile* is a Gram-positive, anaerobic, spore-forming bacillus that can cause diarrhea and colitis in horses, as well as many other mammalian species [90]. Transmission occurs via the fecal–oral route of either the vegetative organism or resistant endospores [94]. Endospores can persist in the environment for years and are resistant to oxygen, many disinfectants, and other stressors [91]. *C. difficile* can also be found in the gastrointestinal tract of healthy adult animals [91], and the rate of isolation in clinically normal adult horses ranges from 0 to 4.3 percent [85,95,96,97]. A small subset of colonized horses will develop CDAD, and clinical disease may only become evident when these horses encounter a stressor such as antimicrobials [98]. Up to 62 percent of human neonates may be colonized without any evidence of clinical disease [99], and it is also suspected that a significant number of young foals may be asymptomatic carriers and shedders of *C. difficile* [95].

Although the pathogenesis of *C. difficile* infection is incompletely understood in the horse [91], it is likely similar to the pathogenesis described in humans. In healthy animals, the microbiome rapidly converts primary bile acids into secondary bile acids, but when the microbiome is disrupted, primary bile acids persist (particularly taurocholate [100]) and promote the conversion of *C. difficile* spores to the vegetative state [84]. Once in the vegetative state, the bacterium is able to replicate and produce one of three toxins, Toxin A (TcdA), Toxin B (TcdB) [101], or binary toxin (CDT) [102]. The presence of these toxins is necessary for the induction of clinical disease. Testing can be performed either by PCR targeting the toxin-producing genes [93], or ideally by using an ELISA for A/B toxin formation [90]. Both TcdA and TcdB are cytotoxic and are capable of disrupting Rho-GTPases; this results in the disorganization of the cytoskeleton and subsequent cell death [88,103,104]. In addition to intracellular effects, TcdA and TcdB may also cause the release of substance P from the enteric nervous system, the activation of mast cells, and the release of chemokines, resulting in the recruitment of neutrophils [16]. The binary toxin, CDT, is comprised of two parts—CDTa and CDTb. CDTa is an enzyme, while CDTb facilitates entry of the toxin into the host enterocyte; once inside the host cell, CDTa targets actin, thus disrupting the equilibrium and resulting in cell death [93,103].

*C. difficile* toxins also act directly on ion transporters located on enterocytes, thus causing diarrhea. In a murine model, it was shown that CFTR and CaCC function is inhibited by *C. difficile* infection, indicating that active chloride secretion was not responsible for the diarrhea in this model. Instead, TcdA and TcdB significantly downregulate DRA and SGLT1, and TcdB downregulates NHE3 [57]. Overall, this indicates that *C. difficile* toxins contribute to diarrhea production through the decreased absorption of sodium ions, which provides an osmotic gradient, pulling water into the colonic lumen. Therefore, presumably CDAD in horses is due to a combination of colonocyte death, the recruitment of pro-inflammatory elements, and the inactivation of sodium-absorbing ion transporters on colonocytes.

### 5.2. Clostridium perfringens

*Clostridium perfringens* has been associated with gastrointestinal disease in humans, ruminants, swine, dogs, and horses (particularly foals). The bacterium is a Gram-positive, rod-shaped, aerotolerant anaerobe, and is one of the most rapidly reproducing bacterial species found in the gastrointestinal tract [105]. Disease is induced via the production of up to 16 toxins [106], but enzymes (such as sialidases, collagenases, hyaluronidases) also likely play a role [105]. The species is subdivided into seven types (A to G), based on the production of six major toxins [CPA (alpha), CPB (beta), CPE (enterotoxin), ETX (epsilon), ITX (iota), and NetB (necrotic enteritis-like beta)]. Of these, Type A is the most ubiquitous, and is distributed throughout the environment and the intestine of healthy animals [107]. The bacterium is shed into the environmental via the feces of colonized animals, and it can persist in the environment for years through the production of highly resistant endospores [105]. In support of this, one risk factor for the development of disease associated with *C. perfringens* Type C in horses is the historical presence of livestock on the farm [108]. *C. perfringens* Type C is generally not isolated from the intestinal tract of healthy equines, thus supporting its role in enterocolitis [82].

Because the bacterium is able to produce many combinations of toxins and can be found in the gastrointestinal tract of healthy animals, determining its role in colitis in the adult horse is complicated. The quantity and colonization rates of *C. perfringens* isolated from the horse can shift, even without the onset of clinical signs of disease. For example, in broodmares *C. perfringens* was cultured from the feces of 19% of mares in late gestation, 35.2% of mares at the time of foaling, and 30.2% of mares 1 to 2 months post-foaling. However, none of these mares had evidence of clinical gastrointestinal disease [107]. Another study showed that administering a lysine and methionine supplement to Standardbred racehorses in training induced an increase in fecal *C. perfringens* colony-forming units (CFUs) but was not associated with gastrointestinal disease [109].

There have been several studies showing an association between clinical disease and *C. perfringens* in either the feces or gastrointestinal contents of adult horses [85,110,111,112,113], and Type D may be more prevalent in cases of anterior enteritis in the United Kingdom [114]. It was also demonstrated that when colitis developed in Standardbred racehorses, there was a higher fecal *C. perfringens* CFU count compared to healthy control animals, and there was a positive association between the number of CFUs and the severity of clinical signs [109].

However, it has proven difficult to experimentally induce C. perfringens-associated clinical disease through the administration of the bacterium to healthy adult horses. One study was able to induce mild clinical disease in 9 out of 26 horses administered a broth of *C. perfringens* via nasogastric tube, but only when administered concurrently with molasses [109]. Another study administered CPE intravenously to three ponies, which resulted in severe and fatal colitis [115]. However, this route did not mimic natural infection, nor was the CPE purified prior to administration [116]. Finally, one study induced clinical signs of enterocolitis by administration of a high dose (27–40 mg/kg) of oxytetracycline, and identified a rapid expansion of *C. perfringens* in intestinal contents [117]. One possible method to determine the role of *C. perfringens* in the horse would be to apply *C. perfringens* strains or toxins to specific segments of the equine gastrointestinal tract, as was done with the rabbit ileum [58,118]. Thus, the local intestinal effect can be studied separately from large-scale microbiome and systemic effects.

*C. perfringens* toxins of particular interest in the adult horse, based on epidemiological and antibody studies, include the major toxin CPE [85,119] and the minor toxin CPB2 [120,121]. Unlike many of the other pathogens associated with equine colitis, these toxins do not act directly on existing epithelial cell ion channels, but instead induce diarrhea through the breakdown of tight junctions between epithelial cells and/or pore formation on the lipid bilayer. CPE is able to induce disease by binding to and disrupting specific claudins, which are one of the major components of tight junctions. Furthermore, CPE is able to form cation-selective ion channels on the epithelial cell lipid bilayer [59,122]. These channels allow potassium to leak into the gastrointestinal lumen, while calcium and sodium flow into the epithelial cell [123]. The influx of calcium ions may induce cell death via activation of apoptosis and necroptosis pathways [124]. The mechanism of action of CPB2 is less well described, but it is also a pore-forming toxin [60,61,125] and is associated with antibiotic-associated colitis secondary to gentamicin administration in the horse (see Section 5.4). However, the role of CPE and CPB2 in equine disease has not been established, as the application of CPE- and CPB2-producing isolates to an equine ovarian tumor cell line demonstrated diminished cytotoxicity compared to controls [62]. NetF is also a pore-forming toxin [82]; in order to form a pore, multiple NetF toxin monomers coalesce and bind to sialoproteins on epithelial cell surfaces [126]. It has been associated with clinical disease in foals [127,128], although not in all regions [129], and it has not successfully induced disease in laboratory animals [82].

CPA and CPB toxins have also been implicated in equine disease, as they are the major toxins associated with Type C infection, which has been sporadically reported in adult horses [82]. However, CPA is produced by all types of *C. perfringens*, including Type A, which is frequently isolated from clinically healthy horses and therefore has a questionable role in disease [124]. CPB in piglets has been demonstrated to diffuse into the lamina propria, causing damage to the endothelium and enhancing vascular permeability [63]. CPB is frequently implicated in neonatal foal (<3 days of age) diarrhea [130] as it is inhibited by trypsin, and colostrum contains high levels of trypsin inhibitors. It is theorized that adult horses with pancreatic insufficiency, suffering from malnutrition, or on a diet containing trypsin inhibitors may also be susceptible [113]. CBP2 is also sensitive to trypsin [60]. There is also evidence from a rabbit model that CPB may act synergistically with CPE, which is intermittently produced in Type C infections, to induce clinical disease [58], so perhaps CPE is also relevant in the pathogenesis of Type C infections.

One of the major complications in determining the role of *C. perfringens* toxins in colitis is that there is limited assay availability for toxin identification [110], and toxins degrade rapidly when kept in sub-optimal conditions [131]. The continued optimization of testing protocols for toxins will be essential for elucidating the pathogenesis of this bacterium in equine colitis.

### 5.3. Salmonella spp.

*Salmonella* spp. are an important infectious cause of enterocolitis in the horse, with around 13 percent of colitis cases testing positive on either PCR or fecal culture in a recent study [10]. In a hospitalized equine population, risk factors for *Salmonella* infection include changes in diet, diarrhea at hospital admission, and a fever developed during hospitalization [132]. Other studies have shown that anorexia, antimicrobial administration [10], and intestinal surgery [133,134] predispose equids to the development of salmonellosis [5,135]; these may all induce microbiome changes that also predispose hosts to infection [136]. Based on the human literature, proton pump inhibitors, such as omeprazole, may also increase susceptibility to infection [137]. Clinical disease is associated with the infectious dose [136,138] and serovar [139], as well as host factors, including immune status [139] and other aforementioned risk factors [135]. Clinical signs of salmonellosis are similar to other causes of acute colitis [81], including profuse diarrhea, endotoxemia, and neutropenia [6]. However, a subset of horses present with nasogastric reflux [140] and/or fever, depression, and leukopenia without concurrent diarrhea [6,141].

*Salmonella* spp. are Gram-negative, facultative anaerobic, motile, non-spore forming bacteria that belong to the family Enterobacteriaceae. *S. enterica* is the species associated with diarrhea, and is further divided into 6 subspecies, including *S. enterica*, which is comprised of over 2600 serotypes [139]. The serovar Typhimurium is the most pathogenic and is associated with a higher case fatality rate [142], including in horses [5], but other important serovars in the horse include Agona, Newport, Anatum, and Braenderup [138]. Transmission generally occurs via the fecal–oral route in the horse. The bacterium can persist for prolonged periods in a moist environment [135] and can also be shed by asymptomatic carriers into the environment; these asymptomatic carrier horses are a common source of infection [94,135,143]. There is geographic variation in the distribution of horses infected with *Salmonella*, with warmer and wetter climates and warmer seasons having higher detection rates [14,144].

A variety of virulence factors can explain the differences in pathogenicity, tissue tropism, and host preference seen between *Salmonella* serovars [145]. The genes encoding for these virulence factors can be found in both chromosomal and plasmid DNA [146], and are generally localized to discrete salmonella pathogenicity islands (SPIs) [139]. In humans, 17 SPIs have been identified; the most important is considered the type III secretion system (T3SS), also known as an “injectosome” due to its ability to translocate proteins directly from the bacterial cytoplasm into the host cell [147,148]. T3SS is expressed on contact with host cells [139], and only when it has reached the appropriate site within the gastrointestinal tract. After attachment, it initially injects a *Salmonella* inner protein (SIP), which localizes to the cell membrane and facilitates the injection of more effector proteins [139]. These effector proteins activate Rho-GTPases in the host cell, which subsequently allows the engulfment of the bacterium by the host cell [148]. Effector proteins also induce the NF-KB pathway, which induces a cellular inflammatory response [149]. Genes encoded on SPIs are also responsible for the invasion of other host cells and the downregulation of protective host cytokines [147]. In mice, *Salmonella* tends to invade enterocytes in the distal ileum; it is hypothesized that this is due to changes in the ratios of microbiome-produced short-chain fatty acids [150].

Similar to *C. difficile*, *Salmonella* induces changes in ion transporter expression and activity, leading to diarrhea. After invading enterocytes, *Salmonella* causes the upregulation of chemokines and cyclooxygenase-2, both of which can lead to the upregulation of enterocyte secretory function [16]. In mice, *Salmonella enterica* serovar Typhimurium also causes CFTR to internalize from the apical cell surface to the cytosol, diminishing the activity of CFTR [64]. This effect appears to be serovar-dependent, as infection with both *Salmonella enterica* serovar Typhi [65] and serovar Dublin [66] cause an increased presence of CFTR on the cell membrane. In fact, serovar Typhi uses CFTR as a receptor to gain entry to cells [65].

There is further evidence that *Salmonella* infection in mice decreases the expression of the gene encoding DRA in the colonic epithelium, suggesting a shift from an absorptive to a secretory phenotype among colonocytes [67,151]. DRA is partially responsible for the absorption of chloride; when it is inhibited, chloride remains in the colonic lumen [57]. In addition, *Salmonella* decreases the expression and activity of ENaC [64]. Therefore, *Salmonella* infection potentially simultaneously decreases the absorption of both chloride and sodium ions, providing an osmotic gradient that prevents water absorption and leads to diarrhea. Infection also causes moderate to severe mucosal and submucosal edema, neutrophilic infiltration, and goblet cell depletion [152], all of which contribute further to the decreased absorption of water from the colonic lumen. The effect of the *Salmonella* serovars responsible for equine colitis on chloride secretion and sodium malabsorption has not yet been studied in the equine colon.

### 5.4. Antimicrobial-Associated Diarrhea and the Microbiome

The healthy equine colon and cecum are colonized by a variety of bacteria. The majority of these bacteria (approximately 90 percent) are anaerobes, with the remaining being facultative anaerobes [146]. In addition to the digestion of fiber and production of energy [41,42], the microbiome confers colonization resistance by outcompeting pathogenic bacteria and preventing their establishment [153,154]. The healthy microbiome has developed a number of mechanisms to outcompete pathogens, including bacteriocin production, the depletion of essential nutrients (especially iron), the production of toxic metabolites, competition for adhesion sites, the stimulation of peristalsis, production of secondary bile acids, and induction of host immune responses [155]. Therefore, if the normal flora is disrupted by antimicrobial administration, it stands to reason that it may make the host more susceptible to pathogens such as *C. difficile* and *Salmonella* spp. Alterations in colonic flora induced by antimicrobial administration may persist for up to 30 days [156]. These changes include a significant loss of diversity in bacterial flora [157] and loss of cellulolytic bacterial communities [156], as well as a proliferation of Gram-negative/coliform bacteria that may disrupt the mucosal barrier and lead to endotoxemia [146].

An interesting potential mechanism of antimicrobial-associated diarrhea occurs with strains of *C. perfringens* that contain the gene encoding the CPB2 toxin (*cpb2*). In pigs, most isolates of *cbp2*-positive *C. perfringens* produce the CPB2 toxin. In contrast, in the majority of equine isolates *cbp2* contains a premature stop codon, resulting in a cryptic (non-expressed) gene. However, the in vitro application of gentamicin to these *C. perfringens* isolates results in a reading frame shift and subsequent production of the CPB2 toxin. It is thought that this occurs via the aminoglycoside’s targeting of the 30S bacterial ribosome. Clinically, one equine referral hospital determined that removing gentamicin from their pre-operative antimicrobial protocol was associated with a reduction in the number of cases of typhlocolitis in the post-operative period [158].

The diagnosis of antimicrobial-associated diarrhea is nearly always presumptive [10] and based on the temporal administration of antimicrobials prior to the development of colitis. The time of onset may be as little as 24 h following a single dose of antimicrobials [159], with an average onset of clinical signs of 3.4–5.7 days [160,161]. Of the total number of horses administered antimicrobials, one study showed that around 0.6 percent developed colitis [10]. Essentially all antimicrobials used in equine medicine have been associated with the development of antimicrobial-associated diarrhea, which underscores the importance of a robust microbiome for equine colonic health [87,159,160,161,162,163].

When looking at antimicrobial-associated disease as a percentage of colitis cases, one study showed that 8.3 percent of the horses who develop colitis likely had antimicrobial-associated disease [14,164], while another attributed 28.6 percent of colitis cases to antimicrobial administration [160]. The most common pathogens associated with antimicrobial-associated diarrhea include *C. difficile* and *Salmonella* spp. [10,14,94,157], although these are not identified consistently in all cases. Other factors that affect the normal gastrointestinal flora can also predispose horses to *C. difficile* and *Salmonella* infections, including hospitalization [93,135], changes in diet and management [94], and colic [165].

### 5.5. Neorickettsiosis (Potomac Horse Fever)

In some regions, Neorickettsial infections are a major cause of enterocolitis in the late summer and autumn months [166]. One recent multicenter study showed that detection rates varied between 0 and 31 percent, with the highest rates in Ontario and the Midwestern United States during the summer and fall [14]. Also known colloquially as Potomac Horse Fever, these infections are caused by the Gram-negative, obligate, intracellular bacterium *Neorickettsia risticii,* and in some regions (Ohio and Canada) by the closely related *N. findlayensis* [167,168,169]. The most frequent presenting complaints in horses with Neorickettsiosis included fever, diarrhea, colic, anorexia, depression, and lameness [170,171]. The fever tends to be biphasic, with the first fever (which may only last for a few hours) preceding other clinical signs by several days [146]. Common clinical pathology abnormalities include neutropenia, elevated hematocrit, hypocalcemia, hyponatremia, hyperglycemia, hypochloremia, azotemia, hyperbilirubinemia, and hypoalbuminemia [170]. Laminitis is a common presenting complaint and sequelae, occurring in 36 to 55 percent of infected cases [76,170]. Abortion has also been reported, sometimes months after infection [172]. Recent studies have found that mortality ranges from 27 percent [170] to 32 percent [171]. Subclinical infections are also likely and frequent, based on serosurveys in endemic regions [173].

*Neorickettsia* is regionally distributed; prior to the identification of the causative organism, the disease was known variously as Potomac Horse Fever in Virginia and Maryland [174], Shasta River Crud in Northern California [175], churrío in Brazil and Uruguay [176], and horse cholera in Ontario [177]. States in the Midwestern United States, including Kentucky and Indiana, also see frequent cases [170]. Like other members of the order Rickettsiales, the organism is transmitted primarily by arthropod vectors, specifically the trematode *Acanthatrium oregonense* [173]. *A. oregonense* is found in various life stages within *Elimia virginica* snails, caddisflies, and mayflies, and the intestines of insectivorous bats [173]. The trematode and intermediate host of *N. findlayensis* has not yet been described [173]. Infection most commonly occurs when a horse consumes an aquatic insect that has been parasitized by *N. risticii*-infected *A. oregonense* [178]. The horse may consume the infected insect while grazing, by consuming hay contaminated with insects, or by drinking contaminated water. A common supposition is that lights left on in a barn overnight attract aquatic insects to water troughs and buckets, and subsequently lead to infection [179]. Horses are considered to be accidental or dead-end hosts [178,179]. Once in the horse’s gastrointestinal tract, *N. risticii* is released from its trematode and insect hosts and is able to replicate in colonic epithelial cells [180]. The bacterium is also able to replicate in tissue macrophages, mast cells, and blood monocytes [180].

The sequence of events leading to enterocolitis remains poorly understood [181]. However, when human colonocytes were experimentally infected, microvilli on the apical surface of the cells were severely disrupted; these microvilli contain the majority of sodium channels in the colonocyte [68]. More recent proteomic studies in the human have shown that the NHE3 receptor is found on microvilli in the proximal colon [182,183]. In a study looking at experimentally infected ponies, their colonic tissue exhibited a loss of net absorption of sodium, which the authors suspect may be due to the loss of microvilli [54] via degradation of the colonocyte cytoskeleton [68]. This study also showed that the colonic tissue of *Neorickettsia*-infected ponies may be less sensitive to secretory stimuli, suggesting that the inhibition of absorption may play a larger role in clinical disease than active secretion [54]. Clinically in the horse, the severity of disease correlates with the degree of hypochloremia and hyponatremia in the serum, indicating the importance that the secretion or malabsorption of these ions play in the pathophysiology of disease [75,170].

### 5.6. Equine Coronavirus

Equine coronavirus is a viral cause of outbreaks of acute enterocolitis, often in younger horses after showing or co-mingling with other horses [184]. A significant portion of horses (43 to 46 percent) that are infected can shed the virus asymptomatically [185,186,187]. Draft breeds appear to be predisposed to infection [188], and clinical cases have been reported in North America, Europe, and Japan [184]. Disease incidence seems to peak seasonally in the cooler months [184,189,190,191]. The most common clinical signs include anorexia, fever, and lethargy [53,190,192]. Unlike other causes of equine colitis, diarrhea occurs with a lower frequency—large retrospective studies showed an incidence of 20 [184] to 23 [187] percent of cases; one smaller study observed diarrhea in 80 [190] percent of cases. Colic signs are seen more often than in other causes of colitis, occurring in around 40 percent of clinical cases of equine coronavirus [75,185,187,190]. Rarely, encephalopathic disease has been reported, presumably due to the overgrowth of urease-producing bacteria and increased absorption of intestinal ammonia due to enteric inflammation, leading to hyperammonemia [186,193]. Reported mortality/euthanasia rates range from 3 [187] to 27 [186] percent but generally are much lower than with other causes of enterocolitis.

Equine coronavirus is a betacoronavirus, which is the family of coronaviruses that also includes the human pathogens SARS-CoV, MERS-CoV, and SARS-CoV-2 [189], as well as bovine coronavirus and canine respiratory coronavirus [194]. Coronaviruses are a positive-sense, single-stranded RNA virus [192]. Equine coronavirus has only been detected in horses and donkeys [192], but bovine coronavirus and SARS-CoV-2 can undergo cross-species transmission, including infecting horses [192,195]. Host range and tissue tropism are determined by the virus’s spike protein [189,196]. The transmission of equine coronavirus primarily occurs via the fecal–oral route [197]. Fecal shedding is intermittent [197], and the virus mostly causes disease in adult horses, although it has been detected and can also cause disease in foals [189,198,199,200].

The decreased incidence of diarrhea may be related to the pathogenesis of equine coronavirus. Unlike other pathogens that target colonocytes, equine coronavirus displays tissue tropism for the small intestine. After entering the host cell, the virus is able to replicate within the cytoplasm [201]. Immunohistochemistry has shown the widespread infiltration of equine coronavirus throughout the small intestine [193,202]. In the horse, infection appears to begin in the proximal small intestine, leading to villus blunting and atrophy [203]. The evaluation of tissue from infected horses at necropsy showed necrotizing enteritis, pseudomembrane formation, and significant infiltration of inflammatory cells within the lamina propria and submucosa in the small intestine [189,193]. Cells infected with equine coronavirus may also undergo apoptosis via the caspase-dependent pathway [204]. Together, these effects lead to the malabsorption of fluid and barrier loss along the small intestinal tract [53,193]. In one study, at necropsy the cecum had watery contents and the ventral colon had areas of hemorrhage within the lamina propria, but no other significant gross colonic lesions were apparent [193].

Thus far, no one has identified the ion transporters responsible for diarrhea development in equine coronavirus. SARS-CoV-2, which causes respiratory disease in humans, also has a high incidence of diarrhea (averaging 10% of all cases [205]) and activation of CaCC in the human colon [69]. A related virus (SARS-CoV) has been shown to inactivate ENaC in cell culture [70]. In the small intestine, SARS-CoV-2 may also decrease sodium absorption via the small intestinal amino acid-Na transporter [205]. SARS-CoV-2 also likely increases passive ion secretion via the downregulation of tight junction proteins and epithelial cell damage [206], further contributing to the development of diarrhea. Presumably, some of these mechanisms also occur in equine coronavirus-associated enterocolitis.

### 5.7. Larval Cyathostominosis

Cyathostominosis is an inflammatory disease of the colon caused by the emergence of encysted small strongyle larvae, with a reportedly low incidence in the continental US but higher in Europe and the UK. The clinical disease of larval cyathostominosis occurs if the majority of encysted L3 larvae in a heavily parasitized horse mature to L4 and excyst concurrently. In North America, cases tend to occur in the late fall and early winter [207]. In Europe, this typically happens in the late winter or early spring [208]. The process of exocystosis causes localized inflammation and severe damage to the colonic wall [208], leading to a generalized typhlocolitis [209]. The median age of affected horses is 2 years [210], and in one study, all cases were less than 6 years old [211], suggesting that older animals develop some degree of immunity, although animals of any age may be affected [212]. Most horses present with dullness, inappetence, diarrhea, and weight loss, and many are recumbent [210]. Serum chemistry often reveals hypoalbuminemia and hypoproteinemia [207]. Many horses also display signs of colic, including cecocolic or cecocecal intussusception [211,213]. On ultrasonographic exam, 58% of horses have a thickened colonic wall [210]. Cases that are severe enough to require referral or are recumbent have a low survival rate (45%), and sudden death is not uncommon [210,211]. Unfortunately, many severely affected horses have a history of appropriate anthelmintic treatment [210], and fecal egg counts are frequently negative in the face of larval cyathostominosis [212,214,215,216] since the larvae do not produce eggs, making antemortem diagnosis difficult. Definitive diagnosis requires the observation of larvae on gross and/or histopathological examination [214], although a recently developed CT3-specific IgG(T)-based ELISA shows promise for antemortem testing, specifically as a way to rule out larval cyathostominosis in cases of colitis [71,215].

Small strongyles/small redworms (*Cyathostominae*) are the most common endoparasite of the horse [192]; virtually all horses with pasture access are infected [217]. Over 40 species of *Cyathostominae* can infect horses, and co-infections with 10 to 20 different species are common [209,217,218]. Horses become infected when ingesting the third-stage larvae (L3). These L3 larvae can migrate into and encyst within the wall of the cecum and ventral colon, where they can remain dormant (hypobiotic) for months. As they migrate into the colonic wall, they can cause serious damage to the epithelial surface and reduce nutrient absorption [208]. While encysted, the larvae are relatively resistant to anthelmintics [208], and they undergo maturation into the L4 larval stage. After maturation into L4, they can excyst and migrate back into the intestinal lumen. Over the course of the spring and summer grazing season, the horse many accumulate thousands of encysted L3 larvae. Cases of colitis due to larval cyathostominosis may be increasing in frequency; this is possibly due to the increasing resistance of parasites to anthelminthics [207].

Cyathostome larvae cause diarrhea through the physical disruption of the mucosal surface of the colon and cecum, allowing water and ions to leak into the gastrointestinal lumen. On histological examination, there is generally widespread necrosis and ulceration of the mucosa with the infiltration of neutrophils, as well as vascular thrombosis and hemorrhage [71]. Cyathostomins may also induce an immune response—enterocytes and tuft cells of the gastrointestinal tract sense nematode presence and release alarmins (the cytokines IL-25, IL-33, and thymic stromal lymphopoietin), which activate T helper type 2 cells (Th2) and induce the release of IL-4 and IL-13. The overall response is “weep and sweep”, with the expansion of goblet and tuft cell populations, an increase in mucous production, and an increase in peristalsis [72,219]. However, in addition to the physical damage caused by the emerging L4 larvae, a heavy cyathostomin burden is associated with a significant change in the microbiota. Furthermore, the mucosal damage allows bacterial translocation into the lamina propria and even the systemic circulation. Therefore, it is suspected that dysbiosis and microbiota translocation may also contribute to the development of diarrhea in these horses [71,220,221].

More generally, intestinal nematode infections in animals are associated with secretory diarrhea due to active chloride ion secretion (potentially via CFTR), as shown by Ussing chamber studies. This is as a result of a Type I hypersensitivity reaction, suspected to occur as a protective mechanism by the host [73]. Protozoan intestinal parasites have been shown to induce diarrhea via manipulating the enteric nervous system, as well as inducing active chloride secretion, as is seen with *Giardia duodenalis* and *Cryptosporidium* [222]. *Hymenolepis diminuta*, a tapeworm that infects rats, also affects ion transport via the inhibition of sodium absorption. Interestingly, this tapeworm parasitizes the rat’s small intestine but induces ion transport changes within the colon [223]. Although ion transport changes associated with small strongyle infection in horses have not been studied, it is reasonable to suspect that active chloride secretion and/or decreased sodium absorption may also contribute to the diarrhea seen with L4 emergence, along with physical damage to the mucosal surface and microbiome changes.

## 6. Conclusions

Equine colitis is a frequently fatal and poorly understood disease that affects a significant number of horses annually. Due to the distinctive anatomy of the equine gastrointestinal tract, horses afflicted with colitis rapidly become dehydrated and frequently develop endotoxemia and laminitis. Although colitis from varying etiologies can look very similar clinically, based on what we know about these pathogens in humans and laboratory animals, the underlying cellular mechanisms are likely to vary depending on the specific cause (Figure 2). Because of this, determining the underlying etiology of individual cases of colitis is crucial, as effective targeted therapies may vary widely depending on the responsible pathogen. For example, CFTR channel inhibitors, which can be used as a treatment for secretory diarrhea in humans and dogs [224], may be effective in some specific diarrheal diseases in horses, and ineffective in others.

The limited equine studies focusing on the relationship between pathogens and ion channels hamper the investigation of targeted therapies (Table 1). We know that ion transport distribution along the gastrointestinal tract can vary between species [18], and the horse’s gastrointestinal tract has an especially unique anatomy and function. Therefore, it is difficult to extrapolate findings on the role of ion transporters from other species, making it crucial to investigate these assumptions using an equine-specific model. Furthermore, horses are afflicted by unique pathogens—*N. risticii* has no closely related human pathogen, and different serotypes of *Salmonella* spp. and species of coronavirus infect horses as compared to other animals and humans.

Unfortunately, a significant proportion of equine colitis cases have an undetermined etiology, with over 50% of cases going undiagnosed, particularly in regions without Neorickettsiosis [82,225]. Improving our ability to diagnose clinical cases is a crucial first step in improving outcomes with equine colitis. Possible under-explored etiologies include novel enteric viruses [226,227,228], alternative species or toxins associated with *Clostridial* infection in the horse [225,229], and ciliated protozoa [225,230,231]. To improve outcomes in equine colitis, future studies should explore both as yet unrecognized causes of colitis and ion transport mechanisms altered by infectious pathogens in the horse.

## Data Availability

Data sharing is not applicable.
